# Strengthening Pakistan’s pharmaceutical workforce: from evidence to action

**DOI:** 10.1080/20523211.2025.2600792

**Published:** 2025-12-24

**Authors:** Nadia Bukhari, Bismah Nayyer, Zaheer-Ud-Din Babar

**Affiliations:** a School of Pharmacy, University College London, London, UK; b Department of Population Health Sciences, Faculty of Life Sciences & Medicine, King’s College London, UK; c Department of Clinical Pharmacy & Practice College of Pharmacy, QU Health, Qatar University, Doha, Qatar

## Setting the scene

Globally, pharmacists are increasingly recognised as vital members of the healthcare team. Their contributions extend beyond dispensing to ensuring rational use of medicines, supporting chronic disease management, and delivering essential public health services (Atif et al., 2020; Babar, 2016; Palaian et al., 2022; World Health Organization, 2019). Evidence from diverse health systems demonstrates the clinical and economic value of pharmacists across primary, secondary, and tertiary care (Babar, 2016; Chou et al., 2003; Hwang & Young, 2015; Scahill et al., 2017).

Yet in Pakistan, pharmacists remain marginalised within the health system. Despite more than 40,000 pharmacies operating nationwide, 95% lack a qualified pharmacist (Gulf News, 2019; Pharmacy Council of Pakistan, 2020). This poses major risks for medication safety and rational use. Pharmacists’ invisibility is reinforced by limited roles in policy and practice, outdated regulations, and lack of public awareness campaigns (Hafeez, 1991; Malik et al., 2020; Mumtaz & Shaheed, 1987).

The FIP National Intelligence Report: Pakistan (Bukhari et al., 2023) highlights these systemic barriers and provides a roadmap for reform. It is complemented by insights from a practitioner poll of over 340 pharmacists, revealing frustrations with governance, training, and recognition. Together, these perspectives show both the urgency of reform and the profession’s aspirations (International Pharmaceutical Federation, 2019; UHC2030, 2021).

## Signals from the workforce

Pharmacists participating in the national poll described recurring themes: weak enforcement of existing laws, lack of pharmacist presence in retail, curricula dominated by basic sciences, and scarce clinical placements. Many reported feeling undervalued by patients, physicians, and policymakers, restricted to dispensing roles rather than recognised as clinical experts (Bukhari et al., 2020; Mustafa et al., 2018).

During the COVID-19 pandemic, however, pharmacists stepped forward as frontline providers, ensuring medicine supply chains, patient counselling, and infection prevention (Bukhari et al., 2020; Cucinotta & Vanelli, 2020). This visibility offered a glimpse of what is possible when pharmacists are empowered. The challenge now is translating this momentum into sustained policy reforms.

## Systemic barriers holding back the profession

### Outdated legislation

Pakistan’s Drug Act 1976 and Pharmacy Act 1967 are no longer fit for purpose (Drug Act, 1976; Pharmacy Act, 1967). While the DRAP Act 2012 attempted to modernise oversight, enforcement remains weak. Prescription-only medicines continue to be sold without checks, and there is no consistent mandate for pharmacist presence at every point of sale (Malik et al., 2020).

The pharmacist-to-population ratio set under the Pharmacy Act is outdated at 1:50, and definitions of pharmacy practice fail to reflect current roles in clinical care, public health, and research. Without legislative reform, the profession cannot meet modern healthcare needs (Babar, 2017; Pharmacy Council of Pakistan, 2020).

### Weak professional governance

The Pharmacy Council of Pakistan (PCP) was established to regulate education, practice, and premises, but fragmentation following devolution has weakened its authority. Registration is still paper based, limiting workforce data accuracy and planning capacity (Pharmacy Council of Pakistan, 2020). Without restructuring and digitalisation, PCP cannot provide the leadership needed for reform (International Labour Organization, 2018; World Bank, 2022).

## Shaping future pharmacists

### Curriculum renewal

The PharmD curriculum remains heavily science-oriented, with limited clinical and practice exposure (Atif et al., 2020; Malik et al., 2020; Scahill et al., 2017). Graduates are theoretically strong but ill-prepared for patient-facing roles. Curriculum reform must embed public health, interprofessional education, and structured clinical placements (Moazam & Shekhani, 2018).

### Internships and residencies

Structured practice placements are rare, with limited internships offered by a handful of institutions such as Shaukat Khanum Memorial Cancer Hospital (Skmchandrc, Lahore, 2019). A one-year paid residency (six months in hospital and six in community) should become mandatory for all graduates. This would align with global best practice and equip pharmacists with clinical and communication skills (Chou et al., 2003; Hwang & Young, 2015).

### Competency development

Poll respondents highlighted the need for stronger skills in prescription screening, pharmacovigilance, medication safety, and patient counselling. Embedding these competencies into education and CPD will enhance pharmacists’ confidence and visibility within healthcare teams (Atif et al., 2020; Janzen et al., 2013).

## Beyond the counter: building a modern workforce

### Continuous professional development

In most countries, CPD is mandatory for re-licensure, but in Pakistan it remains voluntary. This contributes to stagnant skills and weak professional recognition (International Labour Organization, 2020). Introducing compulsory CPD, tracked digitally, would strengthen standards and align Pakistan with global trends (Scahill et al., 2017).

### Gender and diversity

Women comprise more than 70% of the health workforce globally and are projected to make up 73% of the pharmacy workforce by 2030 (World Health Organization, 2019). In Pakistan, however, women face barriers including cultural expectations, safety concerns, and limited leadership pathways (Ali, 2013; Ali & Knox, 2008; Gubbins et al. 2015;Khan, 2010; Khan & Naqvi, 2020; Legraien, 2018; Malik, 2020; Mumtaz & Shaheed, 1987; PCSW 2021; Sathar & Kazi, 2000; UN Women Pakistan, 2023).

The National Alliance for Women in Pharmacy (NAWP) under the Pakistan Pharmacists Association is a promising initiative, supporting mentorship and leadership training (Bukhari et al., 2020; UCL School of Pharmacy, 2021). Creating flexible work policies, childcare support, and leadership programmes is critical to retaining and advancing women (International Monetary Fund, 2017; Janzen et al. 2013; Mahmood, 2011; Roomi & Parrott, 2008).

### Advocacy and public recognition

Pharmacists in Pakistan are often seen as dispensers rather than integral members of the health team (Roomi & Parrott, 2008). Advocacy campaigns targeting policymakers, physicians, and the public are essential to reposition the profession. Successful examples from other LMICs, such as Taiwan and Korea, show how expanded pharmacist roles can improve safety and access (Chou et al., 2003; Hwang & Young, 2015).

## From evidence to action: priority areas

The synthesis of evidence highlights five urgent priorities:

1. Regulatory reform – Update the Drug Act and Pharmacy Act to mandate pharmacist presence and expand scope of practice (Drug Act, 1976; Pharmacy Act, 1967).
2. Governance renewal – Strengthen and digitalise the PCP for robust oversight (Pharmacy Council of Pakistan, 2020).
3. Education and training – Modernise the PharmD curriculum and embed structured residencies (Malik et al., 2020; Moazam & Shekhani, 2018; Scahill et al., 2017).
4. Mandatory CPD – Introduce reflective practice and competency-linked re-licensure (International Labour Organization, 2020; FIP, 2019).
5. Gender equity – Enable women through workplace protections, mentorship, and leadership incentives (Ali, 2013; Ali & Knox, 2008; Bukhari et al., 2020; Legraien, 2018; Mahmood, 2011; Malik, 2020; UN Women Pakistan, 2023; Ministry of PDSI, 2022).

## A platform for change

The FIP Workforce Transformation Programme (WTP) provides a structured global framework for workforce planning (FIP, 2019). Pakistan’s collaboration with FIP through the Pakistan Pharmacists Association represents an opportunity to build a needs-based strategy aligned with Workforce Development Goals (International Pharmaceutical Federation, 2020; World Health Organization, 2019).

Equity-focused initiatives, including Equity Pakistan and NAWP, also provide national platforms for gender-sensitive reforms, policy advocacy, and professional development (Ali, 2013; Ali & Knox, 2008; Global Institute for Women’s Leadership, 2025; UCL, 2021). By leveraging these partnerships, Pakistan can accelerate reforms and align with global standards (Center for Global Development, 2022; International Labour Organization, 2020; World Bank, 2022).

## The time for reform is now

Pharmacists in Pakistan remain underutilised due to outdated legislation, fragmented governance, narrow curricula, limited CPD, and weak gender inclusivity. Yet they represent one of the country’s largest health professional groups and are strategically positioned to advance universal health coverage, rational medicine use, and patient safety (World Economic Forum, 2022; World Health Organization, 2019).

The evidence from the FIP Intelligence Report, practitioner poll, COVID-19 response (Gregory, 2023) and international experience is clear: Pakistan must act decisively to empower its pharmacists. Priorities include legal reform, governance strengthening, practice-based education, mandatory CPD, and gender equity initiatives (Ali, 2013; Ali & Knox, 2008; Malik, 2020; UN Women Pakistan, 2023).

This is a pivotal moment. By embedding pharmacists into the heart of the health system, Pakistan can improve outcomes, advance equity, and unlock economic value (International Monetary Fund, Middle East and Central Asia Dept, 2020; Mahmood, 2011). Failure to act will perpetuate inefficiencies and safety risks.

The choice is stark: marginalisation or modernisation. For Pakistan’s pharmacists, and for its patients, the time for reform is now.

## Authors’ contributions

All authors participated in the manuscript’s study design, conceptualisation, drafting, and revision. All authors have read and approved the final text of the manuscript.
